# Anomalous incident-angle and elliptical-polarization rotation of an elastically refracted P-wave

**DOI:** 10.1038/srep12700

**Published:** 2015-08-05

**Authors:** Lin Fa, Yuxiao Fa, Yandong Zhang, Pengfei Ding, Jiamin Gong, Guohui Li, Lijun Li, Shaojie Tang, Meishan Zhao

**Affiliations:** 1 School of Electronic Engineering, Xi’an University of Posts and Telecommunications, Xi’an, Shaanxi 710121, China; 2 Kunlun Energy Company Limited, Hongkong 999077, China; 3 James Franck Institute and Department of Chemistry, The University of Chicago, Chicago, IL 60637, USA

## Abstract

We report a newly discovered anomalous incident-angle of an elastically refracted P-wave, arising from a P-wave impinging on an interface between two VTI media with strong anisotropy. This anomalous incident-angle is found to be located in the post-critical incident-angle region corresponding to a refracted P-wave. Invoking Snell’s law for a refracted P-wave provides two distinctive solutions before and after the anomalous incident-angle. For an inhomogeneously refracted and elliptically polarized P-wave at the anomalous incident-angle, its rotational direction experiences an acute variation, from left-hand elliptical to right-hand elliptical polarization. The new findings provide us an enhanced understanding of acoustical-wave scattering and lead potentially to widespread and novel applications.

Concerning the interior of the Earth, it is a common understanding that the interior is composed of the regular sequences of isotropic thin layers of different properties. When the prevailing wavelength of a seismic wave is larger than the thickness of the individual layers, the sequences of thin layers behave anisotropically, whereas still transversely isotropic^1^,^2^,^3^,^4^. This macroscopically transversely isotropic medium with a vertical axis of symmetry is called a VTI medium^5^. In such a case, the mechanical property of a VTI medium can be described by the elastic stiffness tensor of a hexagonal crystal^3^,^4^,^5^,^6^,^7^. Based on these understandings, the influences of rock anisotropy on polarization, propagation and reflection/refraction of elastic waves have been studied and reported extensively^7^,^8^,^9^,^10^,^11^,^12^, e.g. the polarization direction of an elastic P-wave, which is different from propagation direction; the propagation velocity that is different from phase velocity; and the reflection/refraction coefficients, which vary with respect to the acoustic impendence and anisotropy of media.

The study on reflection of acoustic wave is very important to geophysics; for example, Nedimovic *et al.* analyzed the reflection signature of seismic and aseismic slip on the northern Cascadia subduction interface^13^ and Canales *et al.* discussed the seismic reflection images of a near-axis sill within the lower crust of the Juan de Fuca ridge^14^. Acoustic waves also bear many similarities to optical or electromagnetic waves in propagation, reflection, refraction, and polarization. Grady *et al.* reported linear conversion and anomalous refraction for electromagnetic waves^15^. Genevet *et al.* studied the phenomena of anomalous reflection/refraction of light and its propagation with phase discontinuities^16^. Fa *et al.* predicted the existence of an anomalous incident-angle for an inhomogeneously refracted P-wave^11^.

In this paper, we show that there exists a physically significant anomalous incident-angle for the refracted P-wave. With this anomalous incident-angle, the incident-angle region can be classified into three sections: the pre-critical incident-angle region, the area between the critical incident-angle and the anomalous incident-angle, and the post-anomalous incident-angle region. There are two distinctive phase velocity solutions before and after the anomalous incident-angle. For an inhomogeneously refracted elliptically-polarized P-wave, the anomalous incident-angle will cause an acute rotational-direction variation.

## Results

### Modeling

**Figure 1:**
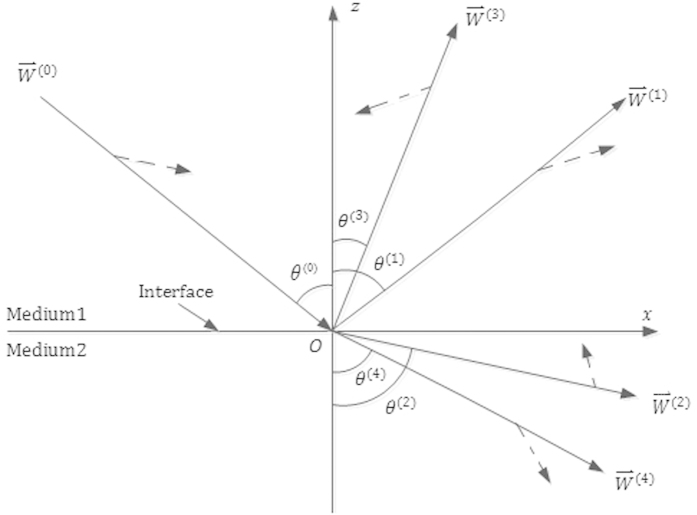
Polarization vector and wave-front normal for incident P-wave and induced waves at the interface. The solid-lines with arrowhead indicate the phase velocity direction and the dashed-lines with arrowhead show the polarization direction; 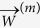 is a displacement and *m* = {0, 1, 2, 3, 4} denotes the incident P-wave, reflected P-wave, refracted P-wave, reflected SV-wave and refracted SV-wave, respectively.

Considering a P-wave propagating in the *x-z* plane impinging on the interface (*x-y* plane) between two VTI media, the system can be described schematically as in Fig. 1.

We performed calculations for two sedimentary rocks with some very well-known physical properties, as reported by geophysicists and given in Table 1 2,11,17. In this paper, we use anisotropic shale (A-shale) as the incidence medium and oil shale (O-shale) as the refraction medium. For this system, by calculation, we found that there is an anomalous incident-angle at 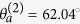.

The elements of the elastic stiffness tensor, related to the anisotropic rock parameters, are given by Thomsen^2^,
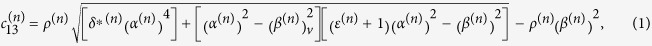

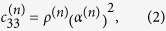

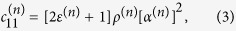

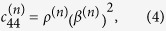

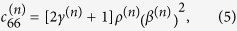


For a harmonic acoustic-field, the wave displacements 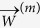 can be written as
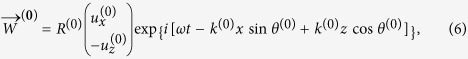

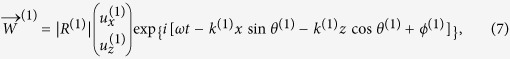

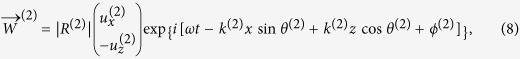

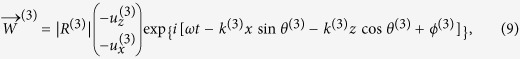

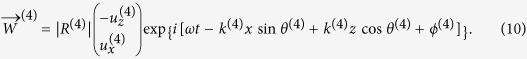


In the equations above, *θ*^(*m*)^ is either an incident-angle or a reflection/refraction angle, and 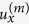 and 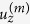 are polarization coefficients; *ϕ*^(*m*)^ is the phase shift for an induced wave relative to the incident P-wave and *ϕ*^(0)^ is defined as 0°; *R*^(*m*)^ is either the reflection or refraction coefficient for each induced wave, and *R*^(0)^ is defined as 1. For the refracted P-wave, the critical incident-angle is denoted by 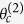, and the anomalous incident-angle is given as 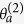.

For the incident-angle range of 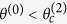, the reflection/refraction coefficients are real (not complex) and *ϕ*^(*m*)^ is 0° or 180°. In the range of 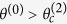, the reflection/refraction coefficients are complex and *ϕ*^(*m*)^ ∈ (−180°, 180°).

### Verification of an anomalous incident-angle

The core existence of an anomalous incident-angle for an elastically refracted P-wave can be confirmed from Snell’s law. Based on the Christoffel equation, the solutions of the phase velocity for the incident wave (P-wave or SV-wave) and the four induced waves are given by^3^,^8^
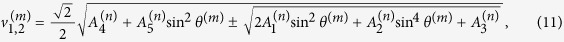
where 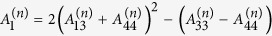

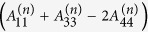
, 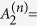

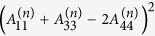
 
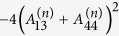
, 
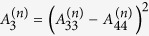
, 
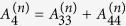
, 
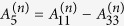
. Denoting the anomalous incident-angle as 
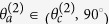
, the phase velocities of the refracted P-wave are 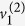 for 
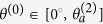
 and 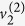 for 
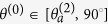
. They abide by Snell’s law such that 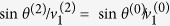 for 
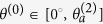
, and 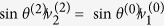 for 
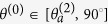
.

The reflection/refraction angles are calculated from the fourth-order polynomials of 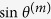
11
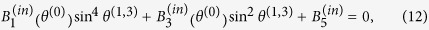

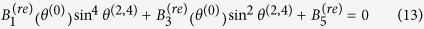
where, 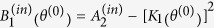, 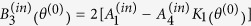, 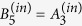 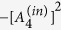, 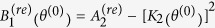, 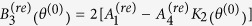, 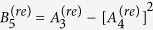, 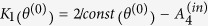, 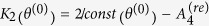, and 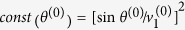. Eq. (13) can be used to calculate the refraction angles and determine the existence of the anomalous incident-angle, denoted by 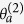.

**Figure 2:**
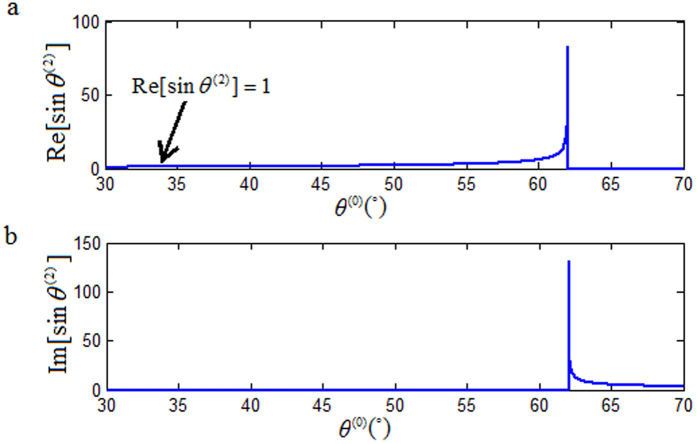
Relationship between sin*θ*^(2)^ and *θ*^(0)^. (**a**) For 
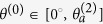
, 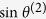 is purely real. (**b**) For 
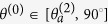
, *sin θ*^(2)^ is purely imaginary. There is an obvious abnormality provided at 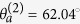.

**Figure 3:**
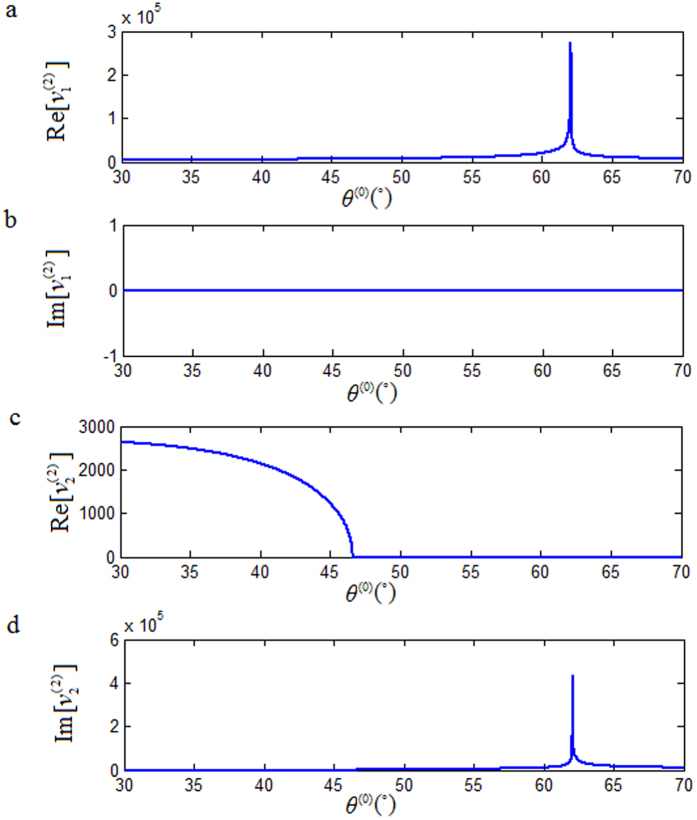
Relationships of both 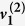 and 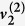 versus *θ*^(0)^. a and b show that 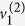 is purely real for *θ*^(0)^ ∈ [0°, 90°], and has a maximum at *θ*^(0)^ = 62.04°; c and d show that 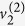 is purely real for 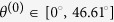 and is purely imaginary for 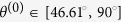. The modulus of 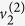 has a maximum at *θ*^(0)^ = 62.04°.

**Figure 4:**
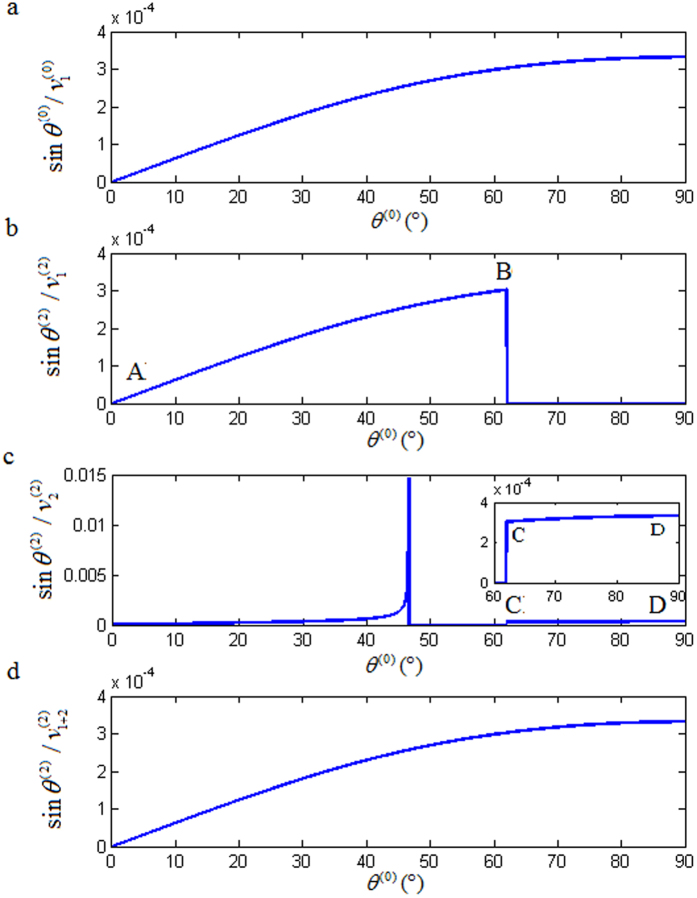
Relationships of 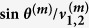 versus *θ*^(0)^. 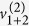
 stands for the phase velocity, 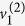, of the refracted P-wave for 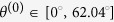 and the phase velocity, 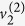, of the refracted P-wave for 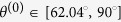.

As shown in Fig. 2a, the value of 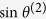 is purely real for 
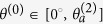
; whereas for 
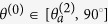
, it is purely imaginary, as shown in Fig. 2b. Figure 3a,b show that for *θ*^(0)^ ∈ [0°, 90°], 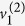 is real (not complex). In Fig. 3c,d, the value of 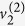 is real for 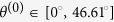 and purely imaginary for 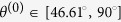. For 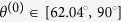, both 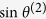 and 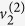 are purely imaginary. Figure 4b,c show that 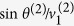 is equal to 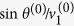 for 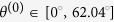 and 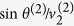 is equal to 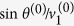 for 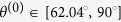. The curve segment 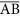 in Fig. 4b plus curve segment 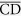 in Fig. 4c forms the curve in Fig. 4d, which is the same as that in Fig. 4a. Here, for 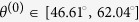, 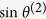 is purely real and 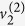 is purely imaginary, so during the plotting of the relationship of 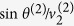 versus *θ*^(0)^, the computer takes the value of 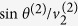 as zero automatically.

These results show clearly that Snell’s law is satisfied only if the phase velocity solution of the refracted P-wave is switched to 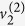 from 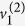 at *θ*^(0)^ = 62.04°. And therefore, there is an anomalous incident-angle of 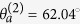. It resides in a region passing the critical incident angle 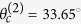, up to an incident angle 90°.

### Verification of elliptically-polarized rotational direction change

Verification of elliptically-polarized rotational direction change can be achieved by invoking the so called energy balance principle. The polarization coefficients for the incident wave and the four induced waves are given by^11^
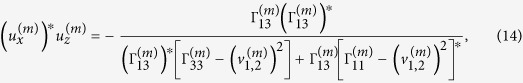
and
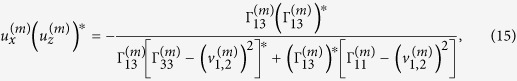
where the definitions of 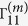, 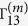 and 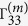 refer to those of Eq. (S2) in “Supplementary material for Anomalous incident-angle and elliptical-polarization rotation of an elastically refracted P-wave”.

From Eqs. (14) and (15) we can obtain the expressions of polarization coefficients for the incident P-wave and the homogenous waves induced at the interface,
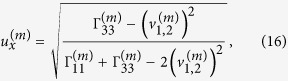

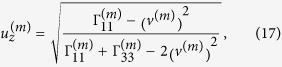


For 
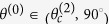
 the refracted P-wave is inhomogenous. Eqs. (14) and (15) provide two sets of solutions for the polarization coefficients (refer to Eqs. (S9) and (S10) in “Supplementary material for Anomalous incident-angle and elliptical-polarization rotation of an elastically refracted P-wave”).
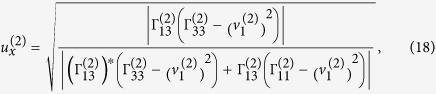

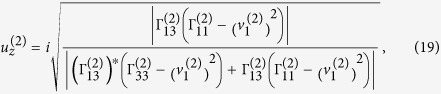
and
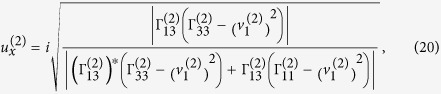

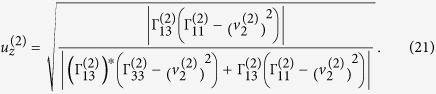


**Figure 5:**
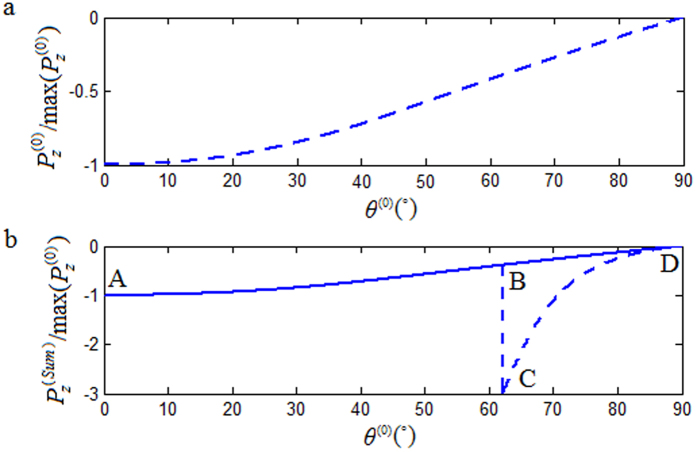
Relationships of 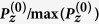 and 
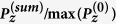
 versus *θ*^(0)^. (**b**) shows that the dashed-line segment 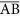 stacks together with the solid-line segment 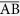 for 
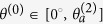
.

An alternative confirmation of the anomalous incident-angle may be achieved by looking at the z-component of Poynting vector, which can be obtained from the reflection/refraction coefficients^11^. Specifically, we look at the normalized z-component, 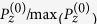, of the incident P-wave. We also look at the normalized real parts of *z*-components from the four induced waves:
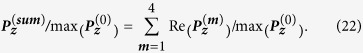


Now, consider the polarization coefficients calculated from Eqs. (16) and (17) as those of the incident P-wave, reflected P-wave, reflected SV-wave and refracted SV-wave for *θ*^(0)^ ∈ [0°, 90°] and the refracted P-wave for 
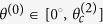
. Meanwhile, Eqs.(18) and (19) provide the polarization coefficients of the refracted P-wave for 
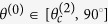
. Then the normalized real parts of z-components of Poynting vectors are plotted dashed-lines in Fig. 5a,b. It shows clearly that, for 
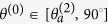
, the real part of 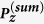 is not identical to 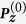. Therefore, it is a violation of the energy balance principle. However, for 
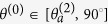
, if we switch the calculation of polarization coefficients from Eqs. (18) and (19) to Eqs. (20) and (21), then the real part of 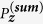 is equal to 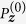, as shown by the solid-line in Fig. 5b, which abides the energy balance principle.

For an inhomogenous refracted P-wave, the *x*-component of the polarization has a lag of 90° with respect to its *z*-component, which is defined as a left-rotational elliptical-polarized wave; otherwise, it is called a right-rotational elliptical-polarized wave. Figure 5 shows that a refracted P-wave is a linearly polarized wave for 
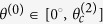
, a left-rotational elliptical-polarized wave for 
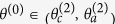
, and a right-rotational elliptical-polarized wave for 
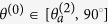
. There is an elliptically-polarized rotational direction change at the anomalous incident-angle 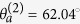.

## Discussion

The current studies of the interface between two VTI media show that there is an anomalous incident-angle 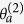 with respect to the refracted P-wave in the area 
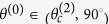
. At such an incident-angle 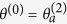, the phase velocity of the refracted P-wave must be switched from 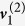 to 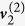 to satisfy Snell’s law. The inhomogeneously refracted P-wave experiences a sudden change from a left-rotational to a right-rotational elliptical-polarization.

**Figure 6:**
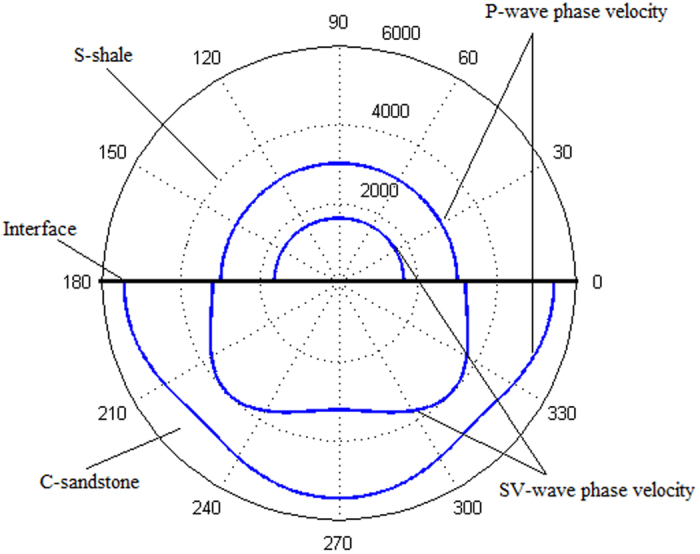
The calculated phase velocity curves for sandstone shale (S-shale) and calcareous sandstone (C-sandstone).

It is worth noting that there is an anomalous incident-angle 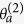 for the refracted P-wave, but no such an anomalous incident-angle *θ*^(4)^ for the refracted SV-wave. As an example, let’s look at the interface between S-shale and C-sandstone. In this case, there are two critical incident-angles, i.e. 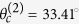 and 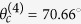. The phase velocity of P-waves in S-shale is smaller than those of P-waves and SV-waves in C-sandstone (see Fig. 6). There is an anomalous incident-angle corresponding to the refracted P-wave at 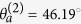. However, even with the second critical incident-angle 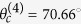 and the refracted SV-wave becoming an inhomogeneous wave for 
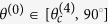
, we have not observed the existence of an anomalous incident-angle 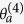 corresponding to the refracted SV-wave.

## Additional Information

**How to cite this article**: Fa, L. *et al.* Anomalous incident-angle and elliptical-polarization rotation of an elastically refracted P-wave. *Sci. Rep.* **5**, 12700; doi: 10.1038/srep12700 (2015).

## Supplementary Material

Supplementary Information

## Figures and Tables

**Table 1:** Anisotropic parameters and elastic constants for A-shale and O-shale.

**Medium**	*α*^(*n*)^ **(m/s)**	*β*^(*n*)^ **(m/s)**	*ρ*^(*n*)^ **(g/cm**^**3**^)	**Thomsen Parameters**			**Elastic constants (GPa)**				
				***ε***^(***n***)^	δ^*(***n***)^	***γ***^(***n***)^	***C***_**11**_	****** 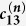	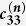	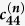	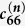
A-shale	2745	1508	2.340	0.103	−0.073	0.345	21.264	6.976	17.632	5.321	8.993
O-shale	4231	2539	2.370	0.200	0.000	0.145	9.397	15.824	42.426	15.278	19.709

The superscript *n* = {*in, re*} donates the incidence medium and refraction medium.
