# Supplementary material for: Anomalous incident-angle and elliptical-polarization rotation of an elastically refracted P-wave
Source: Sci Rep. 2015 Aug 5;5:12700. doi: 10.1038/srep12700 (PMC4525486; doi:10.1038/srep12700)
Supplement: Supplementary Information [file srep12700-s1.pdf]

Supplementary material for Anomalous incident-angle and elliptical-polarization rotation of an elastically refracted P-wave

Lin Fa[1,*], Yuxiao Fa[2], Yandong Zhang[1], Pengfei Ding[1], Jiamin Gong[1], Guohui Li[1], Lijun Li[1], Shaojie Tang[1] & Meishan Zhao[3]

[1] *School of Electronic Engineering, Xi'an University of Posts and Telecommunications, Xi'an, Shaanxi 710121, China*

[2] *Kunlun Energy Company Limited, Hongkong 999077, China*

[3] *James Franck Institute and Department of Chemistry, The University of Chicago, Chicago, IL 60637, USA*

**Derivation of phase velocity solution for elastic wave propagating in VTI medium.**

As illustrated in Fig. 1 in main text, for the case of a P-wave propagating in *x*-*z* plane impinging on a plane interface at *z*=0 between two VTI media with elastic stiffness tensor

$$C^{(n)} = \begin{bmatrix} C_{11}^{(n)} & C_{12}^{(n)} & C_{13}^{(n)} & 0 & 0 & 0 \\ C_{12}^{(n)} & C_{11}^{(n)} & C_{13}^{(n)} & 0 & 0 & 0 \\ C_{13}^{(n)} & C_{13}^{(n)} & C_{33}^{(n)} & 0 & 0 & 0 \\ 0 & 0 & 0 & C_{44}^{(n)} & 0 & 0 \\ 0 & 0 & 0 & 0 & C_{44}^{(n)} & 0 \\ 0 & 0 & 0 & 0 & 0 & C_{66}^{(n)} \end{bmatrix}, \qquad \text{(S1)}$$

and in the absence of body forces, Christoffel equation can be written as [1, 2, 3]

$$\begin{bmatrix} \Gamma_{11}^{(m)} - [v^{(m)}]^2 & 0 & \Gamma_{13}^{(m)} \\ 0 & \Gamma_{22}^{(m)} - [v^{(m)}]^2 & 0 \\ \Gamma_{31}^{(m)} & 0 & \Gamma_{33}^{(m)} - [v^{(m)}]^2 \end{bmatrix} \begin{bmatrix} u_x^{(m)} \\ u_y^{(m)} \\ u_z^{(m)} \end{bmatrix} R^{(m)} = 0, \qquad \text{(S2)}$$

where $\Gamma_{13}^{(m)} = [A_{13}^{(n)} + A_{44}^{(n)}]\sin\theta^{(m)}\cos\theta^{(m)}$, $\Gamma_{11}^{(m)} = A_{11}^{(n)}\sin^2\theta^{(m)} + A_{44}^{(n)}\cos^2\theta^{(m)}$, $\Gamma_{33}^{(m)} = A_{33}^{(n)}\cos^2\theta^{(m)} + A_{44}^{(n)}\sin^2\theta^{(m)}$, $\Gamma_{13}^{(m)} = \Gamma_{31}^{(m)}$, $A_{ji}^{(n)} = C_{jl}^{(n)} / \rho^{(n)}$, the subscripts {*j*, *l*}={1, 2, 3, 4, 5, 6}; $C_{jl}^{(n)}$ is the element in the elastic stiffness tensor and $\rho^{(n)}$ is the density of incidence and refraction media.

Eq. (S2) leads to two uncoupled dispersion relations

$$\{\Gamma_{11}^{(m)} - [v^{(m)}]^2\}\{\Gamma_{33}^{(m)} - [v^{(m)}]^2\} = 0, \qquad \text{(S3)}$$

$$\Gamma_{22}^{(m)} - [v^{(m)}]^2 = 0, \tag{S4}$$

giving the phase velocity solutions of P-wave, SV-wave, and SH-wave, described by Eq. (11) in main text and by

$$v_3^{(m)} = \sqrt{\Gamma_{22}^{(m)}}. \tag{S5}$$

**Polarization coefficients for inhomogenous wave and expressions for inhomogenous refracted P-wave.**

For the incident P-wave and the four induced waves propagating in *x*-*z* plane (see Fig. 1 in main text), Eq. (S2) can be simplified as

$$\begin{bmatrix} \Gamma_{11}^{(m)} - [v_{1,2}^{(m)}]^2 & \Gamma_{13}^{(m)} \\ \Gamma_{31}^{(m)} & \Gamma_{33}^{(m)} - [v_{1,2}^{(m)}]^2 \end{bmatrix} \begin{bmatrix} u_x^{(m)} \\ u_z^{(m)} \end{bmatrix} = 0, \tag{S6}$$

which leads to

$$\frac{u_z^{(m)}}{u_x^{(m)}} = -\frac{\Gamma_{11}^{(m)} - (v_{1,2}^{(m)})^2}{\Gamma_{13}^{(m)}}, \tag{S7}$$

and

$$\frac{u_x^{(m)}}{u_z^{(m)}} = -\frac{\Gamma_{33}^{(m)} - (v_{1,2}^{(m)})^2}{\Gamma_{13}^{(m)}}. \tag{S8}$$

Combination of the normalization condition $u_x^{(m)}[u_x^{(m)}]^* + u_z^{(m)}[u_z^{(m)}]^* = 1$ with Eqs. (S7) and (S8) leads to Eqs. (14) and (15) in main text; here, the superscript ‘ * ’ is the conjugate operator.

From Eqs. (14) and (15) in main text, the modulus of the polarization coefficients can be written by

$$u_x^{(m)}(u_x^{(m)})^* = [u_x^{(m)}(u_z^{(m)})^*]\frac{(u_x^{(m)})^*}{(u_z^{(m)})^*} = \frac{\Gamma_{13}^{(m)}[\Gamma_{33}^{(m)} - (v_{1,2}^{(m)})^2]^*}{(\Gamma_{13}^{(m)})^*[\Gamma_{33}^{(m)} - (v_{1,2}^{(m)})^2] + \Gamma_{13}^{(m)}[\Gamma_{11}^{(m)} - (v_{1,2}^{(m)})^2]^*}, \tag{S9}$$

$$u_z^{(m)}(u_z^{(m)})^* = [(u_x^{(m)})^* u_z^{(m)}]\frac{(u_z^{(m)})^*}{(u_x^{(m)})^*} = \frac{\Gamma_{13}^{(m)}[\Gamma_{11}^{(m)} - (v_{1,2}^{(m)})^2]^*}{(\Gamma_{13}^{(m)})^*[\Gamma_{33}^{(m)} - (v_{1,2}^{(m)})^2] + \Gamma_{13}^{(m)}[\Gamma_{11}^{(m)} - (v_{1,2}^{(m)})^2]^*}. \tag{S10}$$

For the incident P-wave and all homogeneous induced waves, the coefficients $\Gamma_{11}^{(m)}$, $\Gamma_{13}^{(m)}$, $\Gamma_{33}^{(m)}$ and the phase velocity solution $v_{1,2}^{(m)}$ are purely real. So that Eqs. (S9) and (S10) can be simplified as Eqs. (16) and (17) in main text.

The refracted P-wave becomes as an inhomogenous wave for $\theta^{(0)} \in (\theta_c^{(2)}, 90^\circ]$ and

its polarization coefficients are derived in following.

The Poynting vectors of the incident P-wave and the four induced waves propagating on *x*-*z* plane can be written as [2]

$$\vec{P}^{(m)} = -\frac{[\vec{V}^{(m)}]^* \cdot \vec{T}^{(m)}}{2} = -\frac{1}{2}\begin{pmatrix} V_x^{(m)} & 0 & V_z^{(m)} \end{pmatrix}^* \begin{pmatrix} T_1^{(m)} & 0 & T_5^{(m)} \\ 0 & T_2^{(m)} & 0 \\ T_5^{(m)} & 0 & T_3^{(m)} \end{pmatrix} = -\frac{1}{2}\begin{pmatrix} [V_x^{(m)}]^* T_1^{(m)} + [V_z^{(m)}]^* T_5^{(m)} \\ 0 \\ [V_x^{(m)}]^* T_5^{(m)} + [V_z^{(m)}]^* T_3^{(m)} \end{pmatrix}, \quad \text{(S11)}$$

where the symbol '·' is the dot product operator of matrix operation; $\vec{V}^{(m)}$ and $\vec{T}^{(m)}$ are the particle displacement velocity vectors and the stress tensors of the incident P-wave and the four induced waves, respectively.

Substitution of Eqs. (6)-(10) in main text and Eq. (S1) into the relation, $\vec{T}^{(m)} = C^{(n)} : \nabla_s \vec{W}^{(m)}$, of the stress versus the particle displacement [2] leads to the stress-components with respect to the *z*-components of Poynting vectors for the incident P-wave and the four induced waves. Then, bringing these stress-components and the time derivatives of Eqs. (6)-(10) in main text into Eq. (S11) results in

$$P_z^{(0)} = -\frac{\omega k}{2}\{[C_{44}^{(in)}(u_x^{(0)})^2 + C_{33}^{(in)}(u_z^{(0)})^2]\cos\theta^{(0)} + (C_{44}^{(in)} + C_{13}^{(in)})u_x^{(0)}u_z^{(0)}\sin\theta^{(0)}\}, \quad \text{(S12)}$$

$$P_z^{(1)} = \frac{\omega k^{(1)}|R^{(1)}|^2}{2}\{[C_{44}^{(in)}(u_x^{(1)})^2 + C_{33}^{(in)}(u_z^{(1)})^2]\cos\theta^{(1)} + [C_{44}^{(in)}u_x^{(1)}u_z^{(1)} + C_{13}^{(in)}u_x^{(1)}u_z^{(1)}]\sin\theta^{(1)}\}, \quad \text{(S13)}$$

$$P_z^{(2)} = -\frac{\omega k^{(2)}\left|R^{(2)}\right|^2}{2}\{[C_{44}^{(re)}|u_x^{(2)}|^2 + C_{33}^{(re)}|u_z^{(2)}|^2]\cos\theta^{(2)} + [C_{44}^{(re)}(u_x^{(2)})^* u_z^{(2)} + C_{13}^{(re)}u_x^{(2)}(u_z^{(2)})^*]\sin\theta^{(2)}\}, \quad \text{(S14)}$$

$$P_z^{(3)} = \frac{\omega k^{(3)}\left|R^{(3)}\right|^2}{2}\{[C_{33}^{(in)}\left|u_x^{(3)}\right|^2 + C_{44}^{(in)}\left|u_z^{(3)}\right|^2]\cos\theta^{(3)} + [C_{13}^{(in)}u_x^{(3)}u_z^{(3)} + C_{44}^{(in)}u_x^{(3)}u_z^{(3)}]\sin\theta^{(3)}\}, \quad \text{(S15)}$$

$$P_z^{(4)} = -\frac{\omega k^{(4)}\left|R^{(4)}\right|^2}{2}\{[C_{33}^{(re)}|u_x^{(4)}|^2 + C_{44}^{(re)}|u_z^{(4)}|^2]\cos\theta^{(4)} + [C_{44}^{(re)}u_x^{(4)}(u_z^{(4)})^* + C_{13}^{(re)}(u_x^{(4)})^* u_z^{(4)}]\sin\theta^{(4)}\}. \quad \text{(S16)}$$

For $\theta^{(0)} \in (\theta_c^{(2)}, \theta_a^{(2)})$, $\sin\theta^{(2)}$ is a real number which is greater than 1. The relation

$$\sin^2\theta^{(2)} + \cos\theta^{(2)} = 1 \quad \text{(S17)}$$

leads to

$$\cos\theta^{(2)} = \pm i\sqrt{\sin^2\theta^{(2)} - 1}\,, \tag{S18}$$

which is purely imaginary. Therefore, Eq. (8) in main text can be rewritten as

$$\vec{W}^{(2)} = R^{(2)}\begin{pmatrix} u_x^{(2)} \\ -u_z^{(2)} \end{pmatrix}\exp[\alpha_1^{(2)} z]\exp[i\omega(t - \frac{\sin\theta^{(0)}}{v_1^{(0)}}x)]\,, \tag{S19}$$

where $\alpha_1^{(2)} = ik^{(2)}\cos\theta^{(2)} = ik^{(2)}(-i\sqrt{\sin^2\theta^{(2)} - 1}) = k^{(2)}\sqrt{\sin^2\theta^{(2)} - 1}$ is a positive real number and is called the attenuation coefficient of the inhomogeneous refracted P-wave.

For $\theta^{(0)} \in (\theta_a^{(2)}, 90^\circ]$, $\sin\theta^{(m)}$ and $v_2^{(2)}$ are purely imaginary (see Fig. 2 and Fig. 3c,b in main text) and they abide by Snell's law

$$\frac{\sin\theta^{(0)}}{v_1^{(0)}} = \frac{\sin\theta^{(2)}}{v_2^{(2)}}\,. \tag{S20}$$

Therefore, Eq. (S17) can be rewritten as

$$\sin\theta^{(2)} = \pm i\sqrt{\cos^2\theta^{(2)} - 1}\,, \tag{S21}$$

where, $\cos\theta^{(2)}$ is a real number and the sign of $\sin\theta^{(2)}$ is taken positive to keep consistent with the sign of the phase velocity of the inhomogeneous refracted P-wave. Due to $k^{(2)}x\sin\theta^{(2)} = \omega x\sin\theta^{(0)} / v_1^{(0)}$ and $k^{(2)}z\cos\theta^{(2)} = -i\omega z\cos\theta^{(2)} / |v_2^{(2)}|$, Eq. (8) in main text can be rewritten as

$$\vec{W}^{(2)} = R^{(2)}\begin{pmatrix} u_x^{(2)} \\ -u_z^{(2)} \end{pmatrix}\exp[\alpha_2^{(2)} z]\exp[i\omega(t - \frac{\sin\theta^{(0)}}{v_1^{(0)}}x)]\,, \tag{S22}$$

where $\alpha_2^{(2)} = ik^{(2)}\cos\theta^{(2)} = i\omega\cos\theta^{(2)} / v_2^{(2)} = \omega\cos\theta^{(2)} / |v_2^{(2)}|$ is a positive real number and still expresses the attenuation of the inhomogeneous refracted P-wave increasing with $|z|$.

The physical meaning of $\alpha_1^{(2)}$ and $\alpha_2^{(2)}$ are the same. However, the differences between them are: (i) for the expression of $\alpha_1^{(2)}$, $v_1^{(2)}$ is take as the phase velocity solution of inhomegenous refracted P-wave, both $v_1^{(2)}$ and $\sin\theta^{(2)}$ are purely real, and $\cos\theta^{(2)}$ is purely imaginary; (ii) for the expression of $\alpha_2^{(2)}$, $v_2^{(2)}$ is taken as the phase velocity solution of inhomogeneous refracted P-wave, both $v_2^{(2)}$ and $\sin\theta^{(2)}$ are purely imaginary, and $\cos\theta^{(2)}$ is purely real. Eq. (S22) shows that for $\theta^{(2)} \in (\theta_a^{(2)}, 90^\circ]$, the refracted P-wave is still an inhomogeneous wave. Apparently, no matter whether $\theta_a^{(2)}$ exists or not, the refracted P-wave still propagates in *x*-direction

and its amplitude attenuates along the negative $z$-axis direction in terms of the exponential pattern for $\theta^{(0)} \in (\theta_c^{(2)}, 90^\circ]$ which includes the range $\theta^{(0)} \in (\theta_a^{(2)}, 90^\circ]$.

For $\theta^{(0)} \in (\theta_c^{(2)}, \theta_a^{(2)})$, Eq. (S14) combining with Eq. (S19) leads to

$$P_z^{(2)} = \frac{|R^{(2)}|^2}{2} e^{2\alpha_1^{(2)} z} \{ i\omega\alpha_1^{(2)} (C_{44}^{(re)} |u_x^{(2)}|^2 + C_{33}^{(re)} |u_z^{(2)}|^2) + \omega^2 \frac{\sin\theta^{(0)}}{v_1^{(0)}} [\frac{|\Gamma_{13}^{(2)}|^2 C_{44}^{(re)}}{(\Gamma_{13}^{(2)})^* (\Gamma_{33}^{(m)} - (v_1^{(2)})^2) + \Gamma_{13}^{(2)} (\Gamma_{11}^{(2)} - (v_1^{(2)})^2)} + \frac{|\Gamma_{13}^{(2)}|^2 C_{13}^{(re)}}{\Gamma_{13}^{(2)} (\Gamma_{33}^{(2)} - (v_{1,2}^{(2)})^2)^* + (\Gamma_{13}^{(2)})^* (\Gamma_{11}^{(2)} - (v_{1,2}^{(2)})^2)}]\}, \tag{S23}$$

For $\theta^{(0)} \in (\theta_a^{(2)}, 90^\circ]$, the combination of Eqs. (S14) and (S22) gives

$$P_z^{(2)} = \frac{|R^{(2)}|^2}{2} e^{2\alpha_2^{(2)} z} \{ i\omega\alpha_2^{(2)} (C_{44}^{(re)} |u_x^{(2)}|^2 + C_{33}^{(re)} |u_z^{(2)}|^2) + \omega^2 \frac{\sin\theta^{(0)}}{v_1^{(0)}} [\frac{|\Gamma_{13}^{(2)}|^2 C_{44}^{(re)}}{(\Gamma_{13}^{(2)})^* (\Gamma_{33}^{(m)} - (v_2^{(2)})^2) + \Gamma_{13}^{(2)} (\Gamma_{11}^{(2)} - (v_2^{(2)})^2)} + \frac{|\Gamma_{13}^{(2)}|^2 C_{13}^{(re)}}{\Gamma_{13}^{(2)} (\Gamma_{33}^{(2)} - (v_2^{(2)})^2) + (\Gamma_{13}^{(2)})^* (\Gamma_{11}^{(2)} - (v_2^{(2)})^2)}]\}. \tag{S24}$$

In Eqs. (S23) and (S24), all $\alpha_1^{(2)}$, $\alpha_2^{(2)}$, $C_{13}^{(re)}$, $C_{33}^{(re)}$, $C_{44}^{(re)}$, $\omega^2 \sin\theta / v_1^{(0)}$, $(v_1^{(2)})^2$, $(v_2^{(2)})^2$, $\Gamma_{11}^{(2)}$ and $\Gamma_{33}^{(2)}$ are purely real, whereas $\Gamma_{13}^{(2)}$ is purely imaginary, so the values of $P_z^{(2)}$ in Eqs. (S23) and (S24) are purely imaginary, i.e. the real part of $z$-component of both $P_z^{(2)}$ is equal to zero for $\theta^{(0)} \in (\theta_c^{(2)}, 90^\circ]$.

Suppose that the complex expressions of polarization coefficients for the inhomogeneous refracted P-wave are

$$u_x^{(2)} = a_1^{(2)} + ib_1^{(2)}, \tag{S25}$$

$$u_z^{(2)} = a_3^{(2)} + ib_3^{(2)}, \tag{S26}$$

where $a_1^{(2)}$, $b_1^{(2)}$, $a_3^{(2)}$ and $b_3^{(2)}$ are real number. Substitution of Eqs. (S25) and (S26) into Eqs. (S23) and (S24) leads to

$$a_1^{(2)} a_3^{(2)} + b_1^{(2)} b_3^{(2)} = 0. \tag{S27}$$

From Eqs. (S25)-(S27), we can obtain the relations:

$$u_x^{(2)} [u_z^{(2)}]^* = (a_1^{(2)} + ib_1^{(2)})(a_3^{(2)} - ib_3^{(2)}) = i(b_1^{(2)} a_3^{(2)} - a_1^{(2)} b_3^{(2)}). \tag{S28}$$

Eq. (S28) guarantees that if $u_x^{(2)}$ is real, then $u_z^{(2)}$ must be imaginary, or vice verse. Because there is the phase difference of 90$^o$ between $u_x^{(2)}$ and $u_z^{(2)}$ for

$\theta^{(0)} \in (\theta_c^{(2)}, 90^\circ]$, the refracted P-wave is inhomogeneous oval-polarization wave. For the inhomogeneous oval-polarization refracted P-wave, if we take $u_x^{(2)}$ as a real number and $u_z^{(2)}$ as an imaginary number, then its polarization coefficients is expressed by Eqs. (18) and (19) in main text, and if we take $u_x^{(2)}$ as an imaginary number and $u_z^{(2)}$ as a real number, then the polarization coefficients are given by Eqs. (20) and (21) in main text.

**Supplementary references**


[1] Carcione, J. M. *Wave Fields in Real Media: Wave Propagation in Anisotropic, Anelastic and Porous Media* (Elsevier Science Vol. 31, Pergamon, 2001).

[2] Auld, B. A. *Acoustic Fields and Waves in Solids* (Wiley Vol. 1, New York, USA, 1972).

[3] Fa, L., Brown, R. L. & Castagna, J. P. Anomalous post-critical refraction behavior for certain transversely isotropic media. *J. Acoust. Soc. Am.*, **120**, 3479-3492 (2006).
